# Supplementary material for: Getting the message across; a realist study of the role of communication and information exchange processes in delivering stroke Early Supported Discharge services in England
Source: PLoS One. 2024 Mar 8;19(3):e0298140. doi: 10.1371/journal.pone.0298140 (PMC10923427; doi:10.1371/journal.pone.0298140)
Supplement: S1 File — (DOCX) [file pone.0298140.s001.docx]

**Interview Schedule**

IRAS Project ID: 243066

Title of Study: **What is the impact of Stroke Early Supported Discharge? WISE study**

Name of Chief Investigator: Rebecca Fisher

Local Researcher(s): Niki Chouliara, Adrian Byrne

**1. Role / Involvement in the service**

1.1 How long have you been involved with the service and what is your role?

1.2. How is your day divided between tasks (patient rehab, travelling, administration)?

**2. Perceived benefits of implementing ESD**

2.1. What are your targets as a service?

2.3 Do you think the service achieves these targets?

2.4. Do you consider the reduction in hospital length of stay a realistic/achievable target? If yes, how do you think this is achieved?

*Its’ been suggested that if a service operates in a fragmented stroke care pathway e.g. disjointed transitions between services/ delays in securing social care packages, ESD managers will consider a more realistic target to invest their efforts and resources in providing quality rehab rather than reducing hospital length of stay.*

2.5. What do you consider the outcomes of the service to be for stroke survivors? Can you give an example?

**3. Model of operation**

3.1. We think that ESD may work differently in different places. What is it about this model of operation that allows you to achieve your targets?

3.2. (If they have seen the service specification) What are in your opinion the key characteristics/ core components of the service specification that allow the team to achieve its targets?

*Research suggests that the service works better for mild/moderate patients. Is that your experience?*

*There is this idea that adopting a flexible approach to eligibility criteria/length of service allows the provision of a service tailored to patients’ needs. What has your experience been? (does this flexibility carry the risk for the service to evolve to a non-evidence based model, failing to demonstrate effectiveness?)*

3.3 (If 7 day working has been adopted). How does it work for you?

*There is, for instance, this idea that if it is not followed by an increase in staffing levels, it could actually lead to lower rehab intensity, what do you think?*

**4. Patient level factors**

4.1. How is the frequency and length of home visits decided for each patient?

4.2. Are certain groups of patients less likely to benefit?

4.3. What is it about the way you work with patients/carers that will help achieve the desired outcomes? *(e.g. managing patients expectations; patients/carers fears and anxieties on returning home; promoting autonomy; information strategy- appropriate format and timing of information provision)*

4.4. How do you promote patients’ adherence to the rehabilitation plan?

**5. Team level factors**

5.1. How do you work together as a team to achieve your targets? Can you bring an example?

5.2. We are interested in the contribution of social workers/rehab assistants/administrators/doctors/ CPs (as appropriate per site) in achieving your targets.

*There is a hypothesis that having a social worker in the team promotes timely discharge and access to personalised budgets. Has this been the case here?*

5.3. What do you consider as effective team working and how is it achieved in your service? (e.g. effective communication, trust and confidence in colleagues’ judgment, strong leadership, shared ethos, enthusiasm)

5.4. What are the main challenges to effective team-working? (e.g. professional silos, lack of understanding of others’ roles, lack of time/resources)

5.5. Do you feel you have the support you need to provide home based rehab/ handle difficult situations in the community?

**6. Organisational factors**

6.1. Do you think the out/in reaching nature of ESD service has any impact on how well it functions?

6.2. Could you comment on the relationship of the service with other services/organisations (e.g. social services)? Does it affect the way the service operates?

*There is this idea that in settings where communication between different services is strained, clinicians working flexibly across boundaries may promote information exchange and collaboration. Has this been your experience? Could you give an example?*

**7. Wider setting/geography**

7.1. Do you think that the outcomes have been the same for patients from different parts of your catchment area?

7.2. Could you comment on the resources (time/staff/travel expenses) required to cover your catchment area?

7.3. We think that ESD may work differently in different places. Can you think of any conditions specific to this area that influence the operation of the service? (Geography/ stroke care pathway/resources)

*One of our theories is that operating within a rural setting will pose additional challenges to the operation of the service and have a negative impact on its effectiveness. Has it worked at all like that here for you? Can you give an example?*

**8. Technology**

How important are effective administration/ technology/ innovation for the smooth operation of the service?

*It has been previously observed that the separate records systems and lack of data sharing practices between services may lead to duplication of assessments/time spent in admin activities at the expense of timely transfers/ time in hands on care. Has this been an issue here?*

**9. Factors contributing to the sustainability of ESD services:**

9.1. If you could change something about this service to make it work more effectively here what would you change and why?

9.2. Does auditing/ monitoring influence your practice and in what way? How useful is this process? (e.g. SSNAP)

9.3. How is change/innovation received and implemented in the service?

**Closing questions**

What would this service look like in an ideal world?

What else do you think we need to know to understand how ESD works here?
