# Supplementary material for: Getting the message across; a realist study of the role of communication and information exchange processes in delivering stroke Early Supported Discharge services in England
Source: PLoS One. 2024 Mar 8;19(3):e0298140. doi: 10.1371/journal.pone.0298140 (PMC10923427; doi:10.1371/journal.pone.0298140)
Supplement: S2 File — (DOCX) [file pone.0298140.s002.docx]

**Focus groups; structure of session**

Title of Study: **What is the impact of Stroke Early Supported Discharge? WISE study**

| **20’** |
| --- |
| **15’** |
| **30’** |
| **25’** |

1. Introduction to the study
2. Purpose of focus group; opportunity for the team to reflect on practice
3. Set group rules (confidentiality/ anonymity)
4. Take informed consent
5. Present group exercise: “*Draw a flowchart showing a patient’s journey from the stroke unit to your service and from your service to any follow-up services. Include any other services you liaise with during this journey”.*
6. What are your targets as a service and as a team? What patient outcomes are you aiming at?
7. Present CMOs on patient outcomes e.g. reduction in hospital length of stay.
8. “*We think that the programme works differently in different places. What are the conditions that help or present obstacles to you achieving your targets? Use the green post-it notes to identify processes/factors that create a receptive context for the successful operation of the service and the red post-it notes to indicate barriers/unfavourable conditions. Consider positioning them along the flowchart you’ve drawn.”*
9. Present CMOs on contextual influences e.g. rurality.
10. We are curious about how you achieve your targets under these conditions. What is it about this model of operation/ the way you work as a team that allows you to achieve your targets?
11. Present CMOs on mechanisms e.g. core components; team working.
12. Close session-thank participants.
