# Supplementary material for: Getting the message across; a realist study of the role of communication and information exchange processes in delivering stroke Early Supported Discharge services in England
Source: PLoS One. 2024 Mar 8;19(3):e0298140. doi: 10.1371/journal.pone.0298140 (PMC10923427; doi:10.1371/journal.pone.0298140)
Supplement: Attachment — Submitted filename: Response to reviewers.docx [file pone.0298140.s003.docx]

Thank you for your helpful comments. Please find below our responses:

| **Reviewer 1** | **Author responses** |
| --- | --- |
| Reviewer: This is an interesting and valuable piece of work. It addresses a much needed area of healthcare given the current conditions in the NHS and the sometimes failure of integrated health and social care services. The background offers thorough and comprehensive context drawing on both literature and policy. The discussion and reflection on the use of Realist evaluation at both the methodological point and in the discussion means that this paper can serve as a useful paper for any new to the process. I thought that this was particularly welcome.  I was expecting to see CMOS set out early on but actually the integration of each set for each finding was very effective. The reader can clearly see how these have been realised or not. The use of quotes peppering this section adds to the reader's understanding (rather than have them set aside in a table as is sometimes the case).  The discussion is extensive. At first I felt perhaps a little too much but actually it is so well informed and makes very specific and pertinent links between the findings and other studies. I liked the way that this was very methodically undertaken. As referred to above, the reflection on RE is a helpful one. However, the later paragraph on normalisation theory, while important, felt like an additive. I wonder whether some mention of this at an earlier point would be useful.  A final important point to make is that I think the findings could clearly have impact on practice, and this makes it an important piece of work. It will also be a valuable paper for anyone undertaking realist evaluation, especially as a novice.  I would highly recommend publication. | We would like to thank the reviewer for their comprehensive and very positive feedback. |
| **Reviewer 2** | **Author responses** |
| Reviewer 2.1.:Very interesting and well written paper. | Thank you for the positive feedback. |
| Reviewer 2.2: . Clarify if a literature or realist review was conducted in advance of the realist interviews. The authors state that the work presented is part of a larger realist method and then state that a literature review (para three methodological framework section) was conducted. Clarify this point in the context of the realist methods / realist study presented in this paper. | Thank you, we have now clarified the type of review and how it contributed to the development of our IPT. |
| Reviewer 2.3: Add some details regarding the realist informed interview questions for the interviews / focus groups. | We have now included the interview schedules as supplementary files. |
| Reviewer 2.4.Methodological framework section, last line, reference the RAMESES guidelines. | Reference number now corrected |
| Reviewer 2.5. Sampling and data collection section, four para, states theory / theories, one IPT tested in this work? | Thank you, this study was part of a wider project which tested further programme theories. However, this paper focuses on one of them and hence we rephrased to avoid confusion. |
| Reviewer 2.6. Clarify that coding was in accordance with the IPT and therefore 'theory coding' as part of the analysis. | In line with recommendations for realist evaluations we followed a retroductive analytic process which included both top-down (theory informed) and bottom-up (inductive) phases. We begun the analysis by assigning codes directly informed by our IPT but we then proceeded to code data that “disconfirmed”, challenged or added further depth and detail to our preliminary theory and could, therefore, inform revisions/refinements. |
| Reviewer 2.7. Table 2, add some more details to the statements for example, building mutual trust for whom / by whom etc, ensure that there is sufficient depth to the statements in the context of the CMO configuration and IPT. Review all tables to ensure sufficient granulation of details. | Table 2 focuses on communication between ESD services and referrers. This information is now repeated in the table to ensure it is clear to the reader. |
